# The Availability of Nutrients from Plant Sources

**Published:** 1914-04-15

**Authors:** 


### THE AVAILABILITY OF NUTRIENTS FROM PLANT SOURCES.

Without attempting to debate the question of the advantages and disadvantages alleged to accrue from the use of foods of animal and vegetable origin, respectively, we are bound to admit, when we analyze the problem of nutrition, that man and animal alike are dependent on plants for a suitable store of nutrients. As a recent writer has expressed the situation: man is at present still a parasite living on the plant kingdom. The final source of human energy is found in plants. In so far as mankind obtains energy by consuming the flesh of the domestic animals, only a fraction of the supply taken by the latter in the plant products which they ingest can ever reach the sphere of usefulness to man. The animals which furnish food to man function as expensive converters of the energy of plants into a form directly available for his uses. Only a very small residue of the total energy-intake of such animals is left in the tissues which they furnish as nutrients to man; the great bulk of what has been consumed has become lost in the processes of animal life during the long periods of growth and maintenance necessary before the animal food-products can be marketed. To appreciate this, one need only consider for a moment what relatively enormous intakes of energy it requires to secure a final few thousand calories in the form of the flesh of cattle for human consumption. A cow eats a liberal plant ration daily during several years before the nutrient products which she furnishes are ready for the market.

It is evident from the foregoing that, considered solely from the standpoint of economy, it would represent a decided advantage if man could utilize more directly the energy which he now commonly secures only after it has been converted by animals, with great incidental losses, into the forms that suit his present preferences, if not his absolute physiologic needs. Our vegetarian friends will at once remind us that it has repeatedly been found quite compatible with health and happiness to provide for human nutritive needs directly and entirely from the plant kingdom. Despite this fact, which must readily be admitted, it is undoubtedly true that the domestic animals still are provided from the plant world with various foods of which man is at present unable to avail himself.

Hitherto the contributions of the vegetable kingdom to the dietary of human beings have consisted essentially of those parts of plants which serve as storage depots—as reserve supplies for subsequent growth. Seeds, roots, tubers and fruits represent the chief types of plant products which find their way into the ration of mankind. These are by no means all well adapted for direct food-service in the human alimentary apparatus; but the progress of science and the industries and the ingenuity of the domestic arts have little by little so improved the form in which such materials as cereals, nuts, etc., are presented for human consumption, that their utilization has become greatly enhanced. Processes for grinding and comminution, the almost indispensable precautions of cooking and baking—all of which we rarely stop to consider as innovations of human ingenuity—are not natural operations; on the contrary, they have been evolved by the genius of man so that he might readily take direct advantage of the energy which, in the cruder forms in which Nature has stored it, some animals are better equipped to utilize. The inherent indigestibility of "raw" starch, as exemplified by the potato, is overcome by cooking; the further comparative resistance of cereal grains to permeation by digestive juices is minimized by milling and subsequent disintegration by cooking. The history of the struggle to wrest energy from the native plant-products without the expensive necessity of calling on the herbivorous animal to act as an intermediator in the process, has been a long one.

There are, however, still other forms of plant structures of which the herbivora make use freely in nutrition, but which mankind has not yet employed with signal success in so far as extracting energy freely from them is concerned. The green parts of plants, rich in such protoplasmic constituents as protein and nuclear material, present the nutrients so completely enclosed in walls of cellulose that they can be utilized only to a small degree and with enormous alimentary waste. We may soften them by cooking and comminute them by mastication, without liberating their foodstuffs to any adequate extent. Man lacks a cellulose-dissolving digestive agent; and hence he cannot depend on the fermentative processes which, in the large cecal reservoirs of herbivorous animals, facilitate the dissolution of the cell-walls of the green fodders.

Friedenthal, in particular, has cherished the belief that it may be possible to accomplish for the so-called green vegetables, rich in plant protoplasm, a sort of comminution which would disintegrate the resistant cell-walls and impermeable structures and render the contents more available for digestion and assimilation by man. The technic of such manipulations, in the case of products like spinach or lettuce or string-beans or any of the large list of green vegetables, involves desiccation and is by no means as easy as might appear at first consideration; for the degree of subdivision called for is both minute and complete, if success is to be attained.

Professor von Bergmann and Dr. F. W. Strauch of the Municipal Hospital in Altona have tested the validity of Friedenthal's scheme by studying the utilization of plant products prepared in the form of impalpable powders by his process. The outcome has been decidedly gratifying and will, we believe, pave the way for useful innovations in the use of vegetable foods. For example, Strauch found the utilization of bean powder fed to men in the form of a puree, far greater than is true of stringbeans served in the usual form. Spinach, carrots, cabbage, etc., similarly prepared, were enjoyed with singular freedom from the troublesome intestinal symptoms which so often follow their use. In such gastric and intestinal diseases as ulcer, colitis and typhoid, in which these "fresh vegetables" are often excluded from the dietary, the comminuted-powder products were employed with impunity and to advantage. The usefulness of these familiar plant-products, so often classed as offenders against the alimentary comfort of those who ingest them, appears to have been augmented by successful innovation in their preliminary treatment. A group of vegetable products has thus been converted from a mechanical agent, serving largely as so-called roughage, into a not unworthy source of nutriment. The efficiency here recorded is rendered the more striking by the report from the Altona clinic that 300 gm. (10 ounces) per day of vegetable powder, equivalent to 3 kg. (6 pounds) of the fresh plant, may easily be assimilated—an amount which in the natural state could not be tolerated. It is not too much to assume that possibly by similar suitable preparation, such plant products as the grasses, which have hitherto been excluded from the dietary of man, may yet be used as direct sources of energy in human nutrition.—*Journal A. M. A.*

